# Ultrasound Molecular Imaging Enhances High‐Intensity Focused Ultrasound Ablation on Liver Cancer With B7‐H3‐Targeted Microbubbles

**DOI:** 10.1002/cam4.70341

**Published:** 2024-10-21

**Authors:** Xialin Xiong, Hang Zhou, Xinzhi Xu, Qihuan Fu, Yujie Wan, Yuting Cao, Rui Tang, Fang Li, Jun Zhang, Pan Li

**Affiliations:** ^1^ State Key Laboratory of Ultrasound in Medicine and Engineering Institute of Ultrasound Imaging The Second Affiliated Hospital Chongqing Medical University Chongqing China; ^2^ Department of Ultrasound Medicine Chongqing University Cancer Hospital Chongqing China; ^3^ Clinical Center for Tumor Therapy The Second Affiliated Hospital, Chongqing Medical University Chongqing China

**Keywords:** B7‐H3, high‐intensity focused ultrasound, liver cancer, microbubble, ultrasound molecular imaging

## Abstract

**Background:**

High‐intensity focused ultrasound (HIFU) is a promising minimally invasive treatment for liver cancer; however, its efficacy is often limited by the attenuation of ultrasonic energy. This study investigates the effectiveness of B7‐H3‐targeted microbubbles (T‐MBs) in enhancing HIFU ablation of liver cancer and explores their potential for clinical translation.

**Methods:**

T‐MBs and isotype control microbubbles (I‐MBs) were synthesized through the conjugation of biotinylated anti‐B7‐H3 antibody and isotype control antibody to the microbubble surface, respectively. Contrast‐enhanced ultrasound imaging was performed to compare the accumulation of T‐MBs and I‐MBs in liver cancer at various time points. The efficacy of T‐MBs in enhancing HIFU treatment was evaluated by measuring the immediate tumor ablation rate and long‐term tumor growth suppression. Additionally, the induced antitumor immune response was assessed through cytokine quantification in serum and tumor tissue, along with immunofluorescence staining conducted on days 1, 3, and 7 post‐treatment.

**Results:**

T‐MBs demonstrated superior liver cancer‐specific accumulation, characterized by higher concentrations and prolonged retention compared to I‐MBs. The combination of T‐MBs with HIFU resulted in significantly enhanced tumor ablation rates and superior tumor growth suppression. Post‐treatment analysis revealed a gradual uptick in cytokine levels within the tumor microenvironment, along with progressive infiltration of antitumor immune cells.

**Conclusion:**

T‐MBs effectively enhance the therapeutic efficacy of HIFU for liver cancer treatment while simultaneously promoting an antitumor immune response. These findings provide a strong experimental foundation for the clinical translation of ultrasound molecular imaging combined with HIFU as a novel approach for tumor therapy.

## 1 Introduction

HIFU, a minimally invasive, image‐guided, adaptable, and repeatable malignancy therapy based on thermal and mechanical ablation, has demonstrated safety and efficacy in numerous clinical trials for liver cancer patients [1, 2, 3]. It can be combined with radiotherapy, immunotherapy, and transcatheter arterial chemoembolization and also serves as a bridge therapy for liver transplantation [4, 5, 6]. Beyond its physical effects of inducing coagulation necrosis and apoptosis within tumor tissue, HIFU generates tumor cell fragmentation, exposing tumor antigens. Notably, HIFU has been shown to activate antitumor immune cells, including dendritic cells (DCs), M1 macrophages, and cytotoxic T cells, fostering an immune response against tumors [7, 8, 9]. Moreover, studies indicate that HIFU can synergize effectively with systemic immunotherapy [10]. However, the efficacy of HIFU is compromised when tumors are located deep or possess abundant blood supply, leading to attenuation of ultrasonic energy. In such cases, higher ultrasonic output power is necessitated to maximize tumor elimination, posing a concomitant risk of damage to normal tissue [11, 12, 13]. Furthermore, ultrasound, the primary modality for guidance and monitoring during HIFU, encounters challenges in compromised imaging quality.

Enhancing HIFU ablation efficacy by increasing ultrasonic energy deposition in the targeted region is pivotal. Ultrasound contrast agents (UCAs), including microbubbles, nano or micron particles with a liquid–gas phase‐change core, have demonstrated promise as synergists to improve HIFU efficacy. While some studies have showcased their effectiveness in laboratory settings, clinical translation remains limited [14, 15]. Microbubbles, with gas‐filled cores, offer substantial potential to enhance HIFU treatment. They not only lower the cavitation threshold induced by ultrasound, enhancing the ablation effect, but also reduce the required output power for HIFU treatment. Additionally, serving as contrast imaging agents, microbubbles provide precise visualization of lesion boundaries, enabling guided therapy with improved imaging capabilities for deep‐seated lesions.

However, currently, available clinical UCAs such as SonoVue, Levovist, and Sonazoid are effective primarily in arterial phase imaging of hepatocellular carcinoma with a short duration, insufficient for supporting the entire HIFU treatment process [16, 17, 18]. Sonazoid has a longer residence time in the liver, whereas it primarily accumulates in liver parenchyma to enhance the Kupffer phase imaging [19], potentially exacerbating therapeutic ultrasound attenuation and posing a risk of premature defocusing, thus impairing HIFU ablation and causing unwanted damage to normal tissues [20]. Efficient accumulation of microbubbles within tumor tissues for an adequate duration could circumvent these challenges.

**FIGURE 1:**
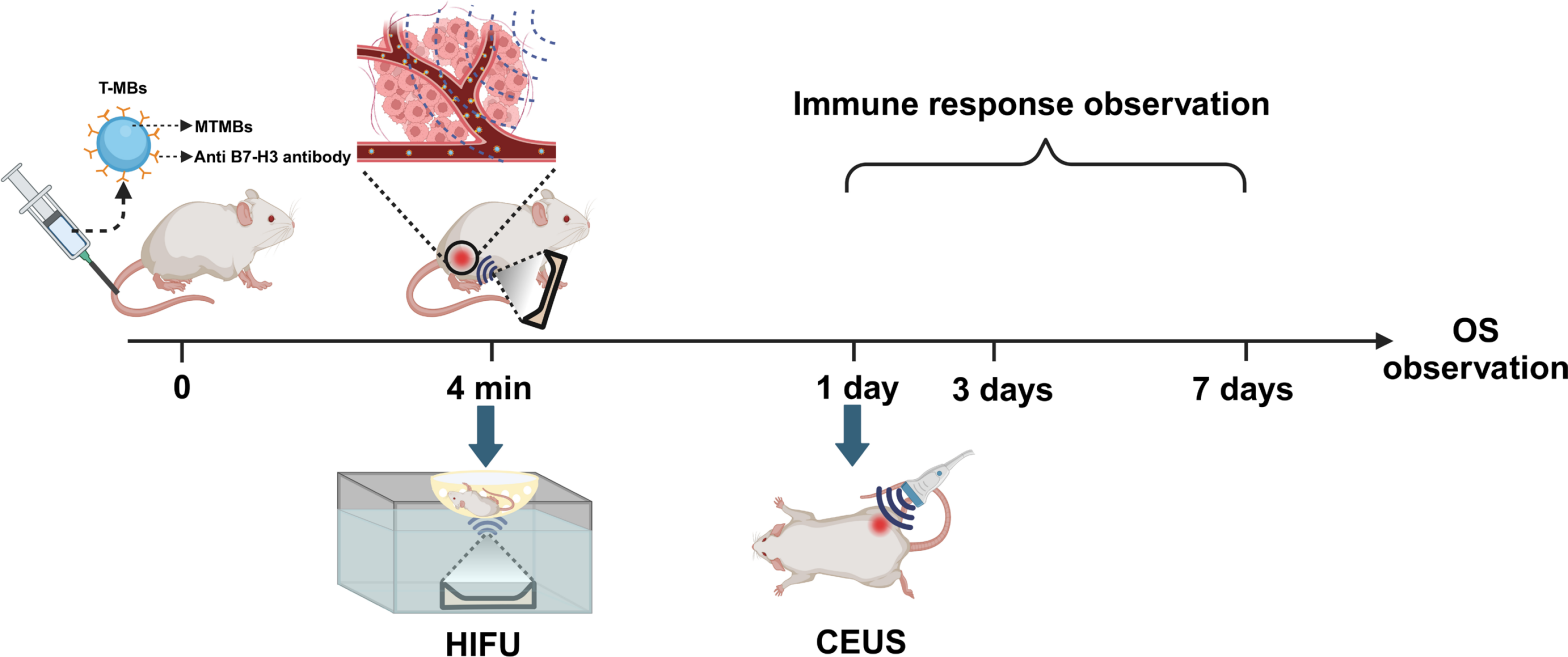
Experimental timeline for B7‐H3 targeted microbubbles enhancing HIFU treatment in mice with subcutaneous liver cancer. Targeted microbubbles (T‐MBs) were synthesized by conjugating anti‐B7‐H3 antibody with platformized multi‐target microbubbles (MTMBs). High‐intensity focused ultrasound (HIFU) was applied at the peak of the targeting period, 4 min after T‐MBs intravenous injection. Contrast‐enhanced ultrasound (CEUS) was conducted 1 day after HIFU treatment to evaluate the immediate tumor ablation rate.

Ultrasound molecular imaging (USMI), an emerging ultrasonography technique enhanced by UCAs with ligands targeting to cancer‐specific molecular markers, has demonstrated value in various human clinical trials [21, 22, 23]. USMI enables specific aggregation of microbubbles in tumors, providing the potential for specific imaging and synergism with HIFU therapy. Recently, a kind of platformized precursor of multi‐target microbubbles (MTMBs) has been developed on an industrial scale. It can be easily transformed into targeted microbubbles by conjugating ligands specific to the target receptors, making it more amenable to translation. While this platform has been widely used in preclinical studies for various cancers, such as breast cancer, gastrointestinal disease, and renal tumor [22, 24, 25, 26], there is a notable absence of reported studies on ultrasound targeted imaging‐enhanced HIFU treatment for liver cancer. In this study, we conduct preclinical research utilizing MTMBs to prepare targeted microbubbles (MBs) against the B7‐H3 molecular marker, an immune checkpoint highly expressed in the endothelial cells of neovascularization in malignant tumors [27, 28]. These MBs will be utilized for ultrasound contrast imaging and enhanced HIFU ablation on liver cancer. We aim to validate its efficacy and provide additional experimental evidence for the clinical translation of USMI enhancing HIFU treatment. The experimental timeline is shown in Figure 1.

## 2 Materials and Methods

### 2.1 Mouse Tumor Model

Animal experimental procedures strictly adhered to the guidelines set by the Animal Ethics Committee of the Second Affiliated Hospital of Chongqing Medical University (Ethical approval number: (2023) 594). The H22 liver cancer cell line was cultured in RPMI1640 medium supplemented with 10% fetal bovine serum and 1% penicillin–streptomycin under controlled conditions (5% CO_2_, 37°C). Subcutaneous liver cancer tumors were successfully established in male Balb/c mice (Ensiweier Biotechnology, Chongqing, China) aged between 6 and 8 weeks. H22 cells in the logarithmic growth phase were harvested, centrifuged, and resuspended in PBS. Each mouse received a subcutaneous injection of 1 × 10^6^ cells into the root of the right hind leg. Approximately 10 days later, when the tumor size reached approximately 80 mm^3^, mice were randomly assigned to groups for either ultrasound imaging or treatment.

### 2.2 Targeting Microbubbles Preparation

The MTMBs (Target‐Ready MicroMarker, Bracco, Geneva; Visualsonics, Canada), which are lyophilized microbubbles with a lipid‐based outer shell that contain polyethylene glycol, phospholipids, and fatty acids, and filled with perfluorobutane (C4F10), and with a mean diameter of 1.5 μm (range, 1–2 μm), were reconstituted in 1 mL sterile saline (0.9% sodium chloride) to prepare targeted microbubbles. Then, 6 μg of biotinylated antibodies was incubated with 5 × 10^7^ streptavidin‐coated microbubbles for 10 min at room temperature. B7‐H3‐targeted microbubbles (T‐MBs) and isotype control microbubbles (I‐MBs) were synthesized by conjugating microbubbles with biotinylated anti‐B7‐H3 antibody (M3.2D7, eBioscience; San Diego, CA) and isotype control antibody (eBR2a, eBioscience; San Diego, CA), respectively. The amount of antibody added was chosen based on previous studies. The mean number of attached antibodies per square micrometer of microbubble surface was approximately 7600 for both types of bubbles [29]. The conjugation efficiency of microbubbles to anti‐B7‐H3 was determined by flow cytometry (TCS SP8, Leica, Wetzlar, Germany).

### 2.3 In Vivo Ultrasound Molecular Imaging

Ultrasound imaging was conducted on Day 10 after tumor inoculation. Ten mice were divided into two groups, each receiving an intravenous administration of a 50 μL microbubble suspension: T‐MBs and I‐MBs, respectively. The tumors in mice were imaged using a clinical ultrasound system (Aplio i800, Canon, Tokyo, Japan) equipped with the PLI‐1205BX transducer, utilizing both B‐mode (frequency: 6.0 MHz, MI 0.04) and contrast‐enhanced mode (frequency: 5.5 MHz, MI 0.09) on the largest cross‐section. Following the injection of microbubbles, an 8‐min observation period was conducted, and a time intensity curve (TIC) was generated based on the quantitative analysis of the contrast signal. Contrast‐enhanced ultrasound (CEUS) static images from T‐MBs and I‐MBs were captured and compared at different time points. The intensities of ultrasound images were measured using the DFY software (Chongqing Medical University, China). Additionally, CEUS imaging on the liver was also observed in the mice.

### 2.4 Optimization of Acoustic Power

A HIFU system (JC‐200, Chongqing Haifu Medical Technology, China), comprising therapeutic and diagnostic ultrasound units, was employed in this study. The therapeutic transducer featured a focal length of 140 mm, a diameter of 220 mm, and an operating frequency of 0.94 MHz. The focal region measured 9.8 mm along the beam axis and 1.3 mm in the transverse direction. A diagnostic transducer, with center frequencies ranging from 3.5–5 MHz, was centrally positioned within the therapeutic transducer.

To observe the immediate ablation effect of HIFU and optimize the acoustic power, tumor‐bearing mice were randomly assigned to six groups and subjected to different treatments, including HIFU alone (70 W, 90 W, 110 W) and I‐MBs + HIFU (70 W, 90 W, 110 W). After anesthetizing the mice with 1% pentobarbital sodium, the tumors were sonicated at different acoustic intensities with an exposure duration of 2 s under ultrasound guidance. The ablation effect was confirmed and comparatively analyzed based on grayscale changes (GSCs) during the treatment. The GSCs primarily reflect the differences in the echo signals of the tumor area before and after HIFU treatment. To minimize heat‐induced complications, the lowest acoustic power causing GSCs representing tissue coagulation necrosis was optimized for subsequent experiments.

### 2.5 Therapeutic Efficacy of MBs‐Enhanced HIFU Ablation

To validate the therapeutic efficacy of MBs‐enhanced HIFU, tumor‐bearing mice were allocated to four groups: Control, HIFU alone, I‐MBs + HIFU, and T‐MBs + HIFU. To underscore the enhancement effect of T‐MBs in augmenting HIFU ablation efficiency, sonication with optimized acoustic power was performed in the fourth minute after MBs injection when T‐MBs were assumed to have fully bound to tumor neovascular endothelium and accumulated in the tumor area. Sonication focus was sequentially traversed at three adjacent, nonoverlapping sites, covering the entire tumor. Mice in the control and HIFU alone groups were injected with an equivalent volume of saline.

On the first day after treatment, CEUS imaging was conducted with the injection of 50 μL Sonovue (Bracco, Milan, Italy) microbubbles to assess tumor blood perfusion. Using ImageJ software, the long (*L*) and short (*S*) diameters of both the tumor and the non‐enhanced regions within the tumor were measured on ultrasound images. The volumes of the tumor and the non‐enhanced regions were calculated using the formula: Volume (mm^3^) = *L* × *S* ^2^ × π/6. The ablation rate (%) is calculated as follows: (volume not enhanced after treatment−volume not enhanced before treatment)/(tumor volume−volume not enhanced before treatment). Subsequently, tumors were fixed with 4% paraformaldehyde and subjected to hematoxylin and eosin (H&E) staining, proliferating cell nuclear antigen (PCNA), and terminal deoxynucleotidyl transferase dUTP nick end labeling (TUNEL) immunofluorescence staining.

The long (L) and short (S) diameters of the tumor were measured using vernier calipers every 2 days for a continuous period of 2 weeks. The tumor size was estimated using the formula: *L* × *S* ^2^ × π/6. Survival observations were conducted for all mice until natural death occurred or, if any tumor reached a size of 2000 mm^3^ or larger, the mice were humanely sacrificed.

### 2.6 Monitoring of the Immune Response

To investigate the HIFU‐induced immune response, mice were sacrificed on Days 1, 3, and 7 after treatments with Control, HIFU alone, I‐MBs + HIFU, and T‐MBs + HIFU, respectively. On Day 1, immunofluorescence was employed to determine the expression of heat shock protein 70 (Hsp70) and calreticulin (CRT) in tumor tissues. Primary monoclonal antibodies (mAbs) used for immunofluorescence assays included anti‐Hsp70 (AF300494, AiFang Biological, Hunan, China) and anti‐CRT (AF300381, AiFang Biological, Hunan, China).

On Days 1, 3, and 7, ELISA kits (Jingmei Biotechnology, Jiangsu, China) were used to quantify cytokine levels in mouse serum and tumor tissue, including interferon‐gamma (IFN‐γ), tumor necrosis factor‐alpha (TNF‐α), Interleukin‐6 (IL‐6), and Interleukin‐10 (IL‐10). Immunofluorescence staining of tumor tissue sections was performed with mouse‐specific fluorochrome‐conjugated monoclonal antibodies (mAbs), including CD8 (98941s, CST, Massachusetts, USA), CD86 (#19589, CST, Massachusetts, USA), and CD206 (#124595, CST, Massachusetts, USA) antibodies. The infiltration of CD8+ T cells, M1, and M2 macrophages in tumor tissue was observed. Stained slides were scanned using an upright fluorescence microscope (NIKON ECLIPSE C1, Nikon, Tokyo, Japan), and digital images were viewed using an imaging system (NIKON DS‐U3, Nikon, Tokyo, Japan).

### 2.7 Statistical Analysis

Statistical analyses were conducted using Prism 9 software (GraphPad Software Inc.). Results are expressed as mean ± SD. One‐way ANOVA was utilized for analyses involving three or more groups, followed by a Tukey correction for multiple hypotheses in GraphPad Prism. Differences between the two groups were analyzed using an unpaired *t*‐test, assuming unequal variance. *p*‐values below 0.05 were deemed significant. The probability of survival in animal studies was determined using the Kaplan–Meier method in GraphPad Prism.

## 3 Results

### 3.1 In Vivo Ultrasound Molecular Imaging

**FIGURE 2:**
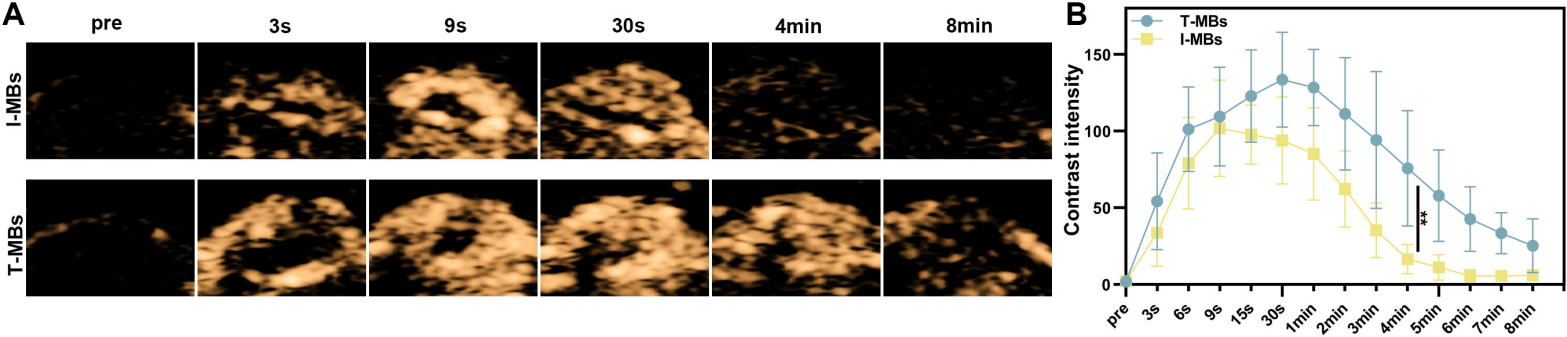
In vivo ultrasound molecular imaging of tumors. (A) Ultrasound molecular imaging of tumors with T‐MBs and I‐MBs in vivo. (B) Grayscale quantitative analysis of A, respectively (*n* = 5, **p < 0.01).

The conjugation efficiency of T‐MBs to the anti‐B7‐H3 antibody was 99.5%, as depicted in Figure S1. Following the injection of T‐MBs or I‐MBs, ultrasound imaging of the tumor exhibited rapid enhancement during the wash‐in phase, typically occurring around 3 s postinjection (Figure 2A). Time intensity analysis (Figure 2B) disclosed discernible differences between I‐MBs and T‐MBs in the time to peak intensity, which were recorded at 9 and 30 s, respectively. Additionally, I‐MBs displayed a more rapid signal decline compared to T‐MBs during the wash‐out phase. Importantly, T‐MBs signals were 4.6 times higher than I‐MBs signals by the fourth minute postinjection, which indicated T‐MBs had been fully conjugated to the highly expressed B7‐H3 sites on tumor neovascular endothelium. This finding is consistent with previous reports [29]. Thus, at this time point, T‐MBs accumulated in the tumor area, providing advantages for subsequent enhanced HIFU treatment. However, no statistical difference in the intensity was observed between the two types of microbubbles in the liver (Figure S2).

### 3.2 Microbubble‐Enhanced HIFU Ablation

**FIGURE 3:**
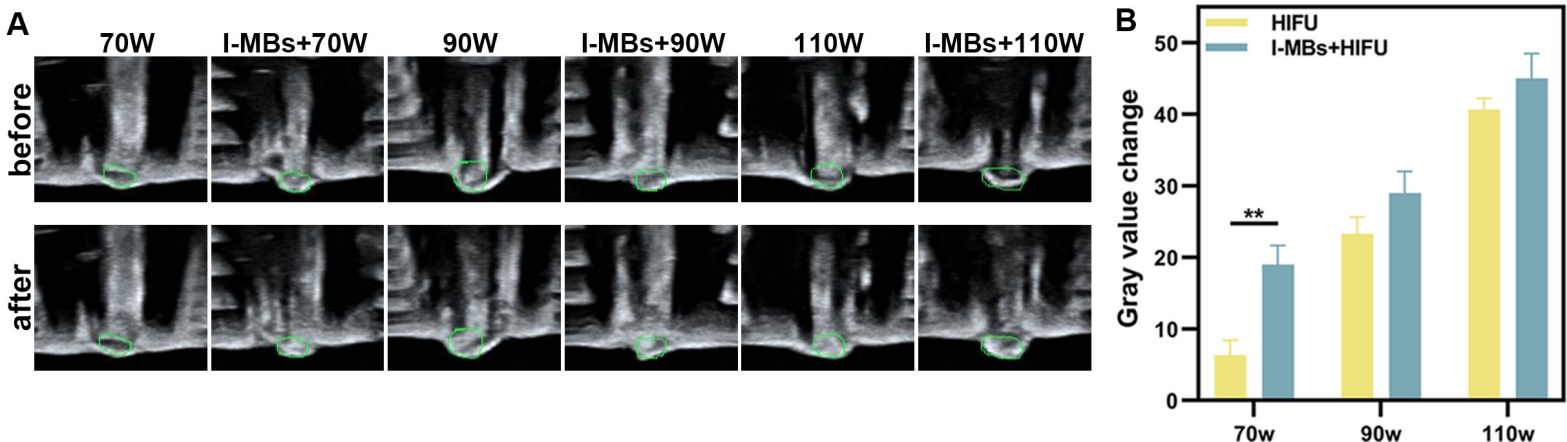
Optimization of Acoustic Power (A) Real‐time ultrasound images before and after HIFU irradiation on tumors at 70, 90, and 110 W for 2 s. (B) The quantitative analyses of A (*n* = 3, **p < 0.01).

The GSCs observed in our study were considered as an indication of successful tumor tissue ablation. As illustrated in Figure 3, there was a noticeable increase in grayscale when HIFU treatments were applied at intensities of 90 and 110 W, even without the injection of I‐MBs. However, only when combined with I‐MBs, HIFU induced a significant GSC at an intensity of 70 W, suggesting the enhanced ablation effect on the tumor due to microbubble use. The use of lower acoustic power during HIFU treatment contributes to improved safety. Based on these findings, we selected an optimized protocol for subsequent microbubble‐enhanced HIFU experiments using a power intensity of 70 W and a duration of 2 s.

**FIGURE 4:**
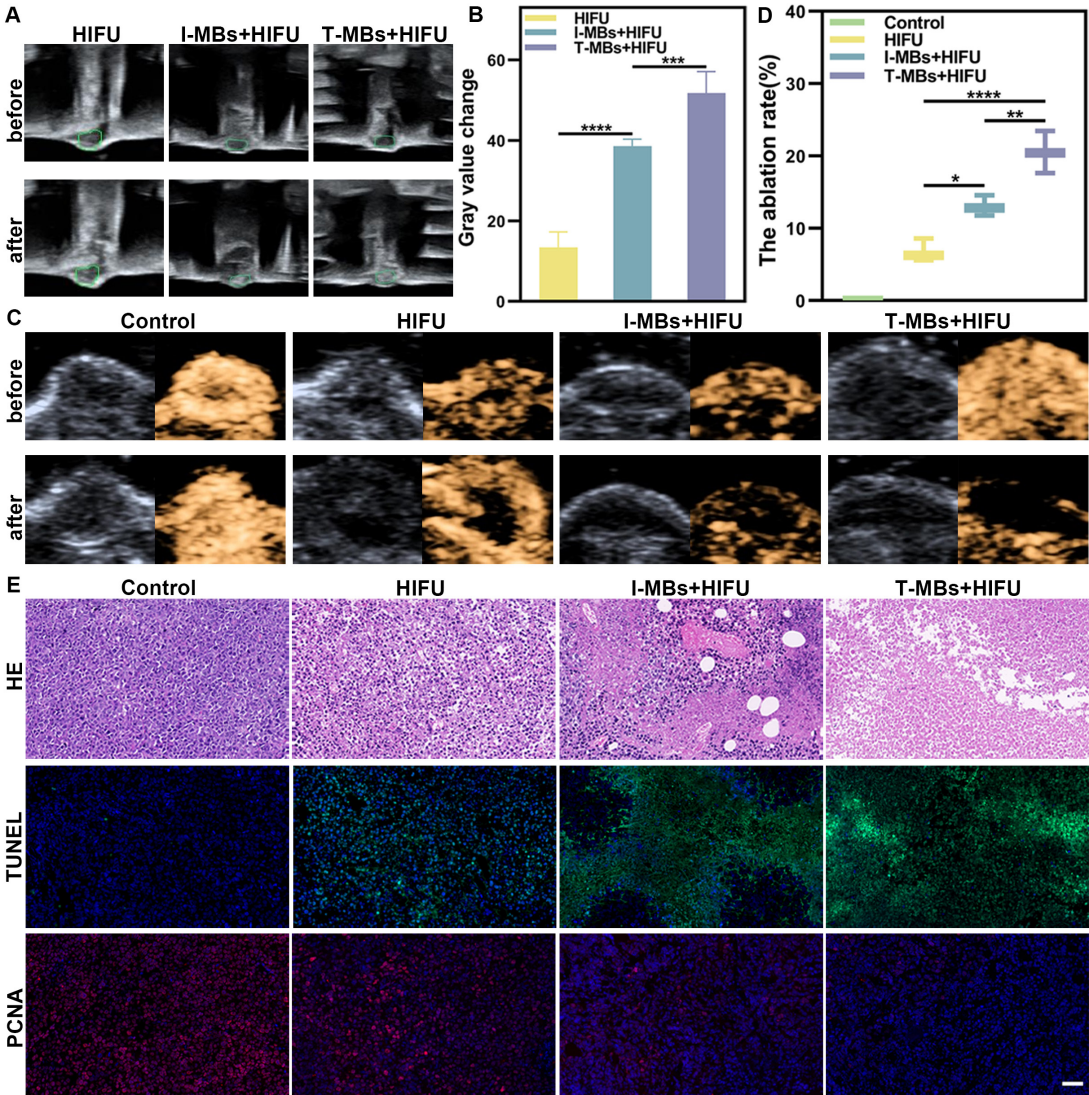
Microbubble‐enhanced HIFU ablation effect. (A) Ultrasound images pre‐ and post‐70 W HIFU irradiation. (B) The quantitative analyses of A (*n* = 5, ***p < 0.001, ****p < 0.0001). (C) In vivo B‐mode and CEUS imaging before and after HIFU irradiation. (D) The ablation rate of C, respectively (*n* = 3, *p < 0.05, **p < 0.01, ****p < 0.0001). (E) H&E, TUNEL (green fluorescence), and PCNA staining images (red fluorescence) of tumors on Day 1 post‐treatment (Scale bar: 50 μm).

We further assessed the capability of T‐MBs to enhance HIFU ablation. Consistent with the results of ultrasound molecular imaging, HIFU treatment was conducted 4 min after microbubble injection. Compared to I‐MBs + HIFU treatment, T‐MBs + HIFU induced more substantial grayscale changes as depicted in Figure 4A,B. Ultrasound imaging revealed that tumors subjected to HIFU exhibited a reduction in blood perfusion and presented varying degrees of filling defects. Notably, T‐MBs + HIFU resulted in the most pronounced defect (Figure 4C). Further quantitative analysis disclosed a significant increase in the non‐enhanced area in the T‐MBs + HIFU group compared to the other groups (Figure 4D). This expanded ablation area underscores the potential of T‐MBs to enhance HIFU ablation efficacy. In Figure 4E, HE staining for the T‐MBs + HIFU group demonstrated a significant disruption in tumor cell structures accompanied by nuclear fragmentation. Analysis of PCNA and TUNEL staining indicated that the red signal associated with cell proliferation in the T‐MBs + HIFU group was the weakest, while the green fluorescence signal related to cell death was the strongest.

### 3.3 T‐MBs Combined With HIFU Treatment for Tumor Growth Suppression

**FIGURE 5:**
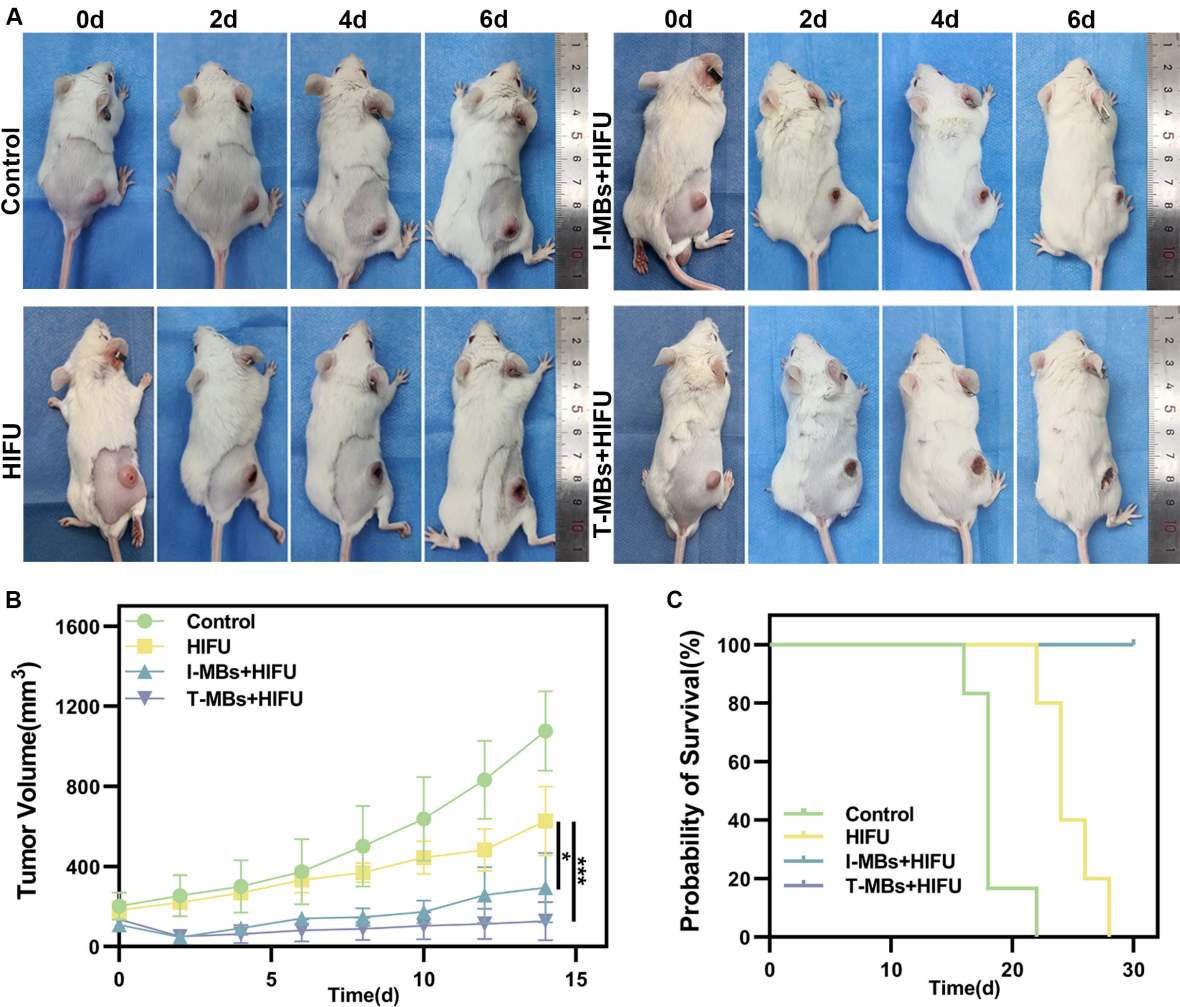
The impact of T‐MBs combined with HIFU ablation on the growth of liver tumors in mice. (A) Images of tumor‐bearing mice on Days 0, 2, 4, and 6 post‐treatment. (B) Growth curve of tumors (*n* = 5, *p < 0.05, ***p < 0.001). (C) Survival curve of mice (*n* = 5).

Based on the immediate ablation effect of HIFU, we further delved into the synergistic antitumor effects of HIFU in combination with MBs. As illustrated in Figure 5A, the tumor following I‐MBs + HIFU treatment exhibited a decelerated growth, while the tumor gradually regressed after T‐MBs + HIFU treatment. The tumor growth curve was depicted in Figure 5B, and a comparison of tumor volumes 14 days after treatment clearly showed that tumors treated with T‐MBs + HIFU were significantly smaller than those subjected to HIFU alone. Throughout the observation period, there was no significant change in the body weights of the mice (Figure S3). The survival curve data underscored a substantial extension in mouse survival following T‐MBs + HIFU treatment (Figure 5C). These findings highlight that T‐MBs, when combined with HIFU, induce a synergistic antitumor effect resulting in prolonged survival in liver cancer tumor models.

### 3.4 Temporal Evolution of Cytokine Response in Serum and Tumor

The evaluation of cytokine level changes in mice at 1, 3, and 7 days after HIFU treatment was depicted in Figure S4, which indicates that 1 day after HIFU therapy, the T‐MBs + HIFU group exhibited a significant surge in serum levels of IFN‐γ, TNF‐α, and IL‐6 compared to the control and HIFU groups. Conversely, IL‐10 experienced a notable reduction. No significant alterations in serum cytokine levels were observed on Days 3 and 7 after HIFU treatment. This suggests that the HIFU treatment likely induced an acute systemic inflammation which gradually subsided over time. Notably, there was a delayed response at the tumor site compared to the swift reaction observed in the serum. One day after HIFU therapy, no significant changes were detected in tumor cytokine levels; however, by Day 3, elevated levels of tumor IFN‐γ, TNF‐α, and IL‐6 were observed in the T‐MBs + HIFU group compared to the control group. On Day 7, this increase persisted for IFN‐γ, TNF‐α, and IL‐6 while IL −10 levels decreased within the T‐MBs + HIFU group (Figure S5). The gradual rise in cytokine levels from Days 1 to 7 after HIFU treatment within the tumor indicates a phased infiltration of the immune response.

### 3.5 T‐MBs Combined With HIFU Therapy Induces Antitumor Immunity

**FIGURE 6:**
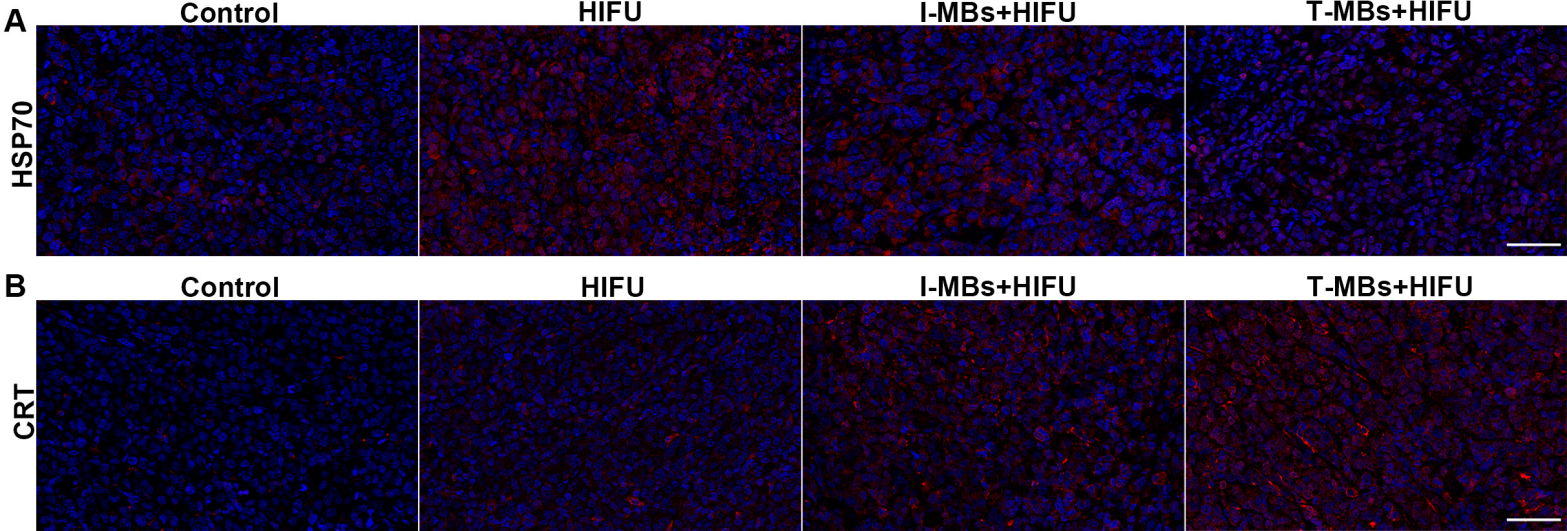
Immunofluorescence analysis of HSP70 (A) and CRT (B) (Scale bar: 50 μm).

**FIGURE 7:**
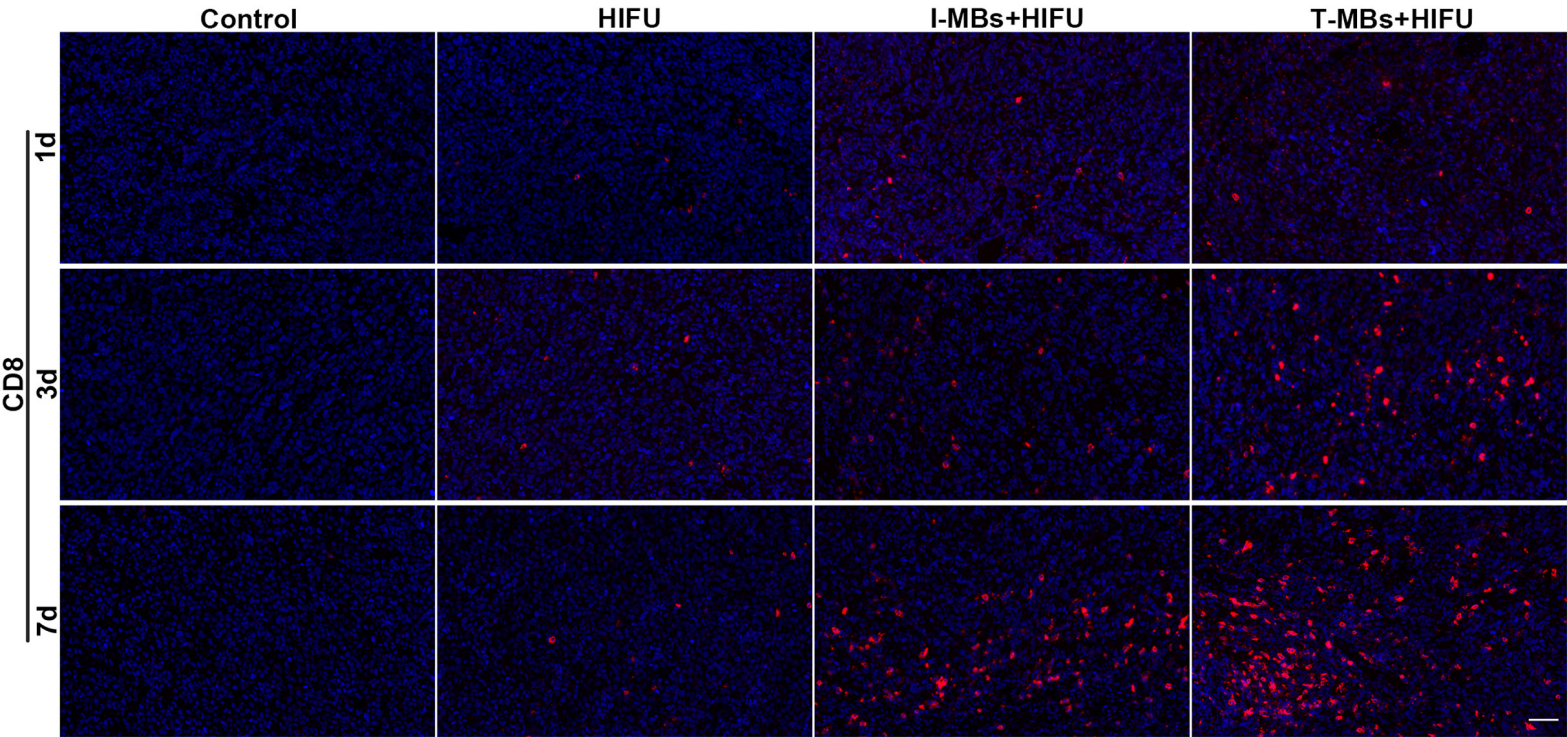
Immunofluorescence analysis of CD8+ T cells (red fluorescence) in tumor tissues (Scale bar: 50 μm).

We further performed immunofluorescence staining on tumor tissues for HSP 70 and CRT expression. And the infiltration of immune cells was also investigated. As depicted in Figure 6A, HIFU treatment alone resulted in a significant increase in Hsp70 fluorescence intensity, indicating a robust thermal effect. In contrast, I‐MBs + HIFU group showed moderate fluorescence intensity, while T‐MBs + HIFU group exhibited the lowest intensity. These findings showed varying degrees of thermal effects induced by HIFU under different conditions, emphasizing the mechanical effect enhanced by T‐MBs. Additionally, substantial CRT expression was observed after treatment with T‐MBs or I‐MBs + HIFU as displayed in Figure 6B, especially in the former group. In contrast, HIFU alone had minimal impact on CRT expression. Simultaneously, a remarkable CD8+ T cell infiltration was found in tumor after treatment with T‐MBs or I‐MBs + HIFU, and increased as time progressed. Moreover, the CD8+ T cell infiltration in T‐MBs + HIFU group was more prominent compared to that in I‐MBs + HIFU group, while only weak CD8+ T‐cell infiltration was observed after HIFU therapy alone (Figure 7). A similar trend was observed for M1 macrophages (Figure S6A). By contrast, the M2 macrophage number in tumor tissue exhibited a decreasing trend after treatment and T‐MBs + HIFU group presented the fewest M2 macrophages (Figure S6B). Taking these results together, we can speculate T‐MBs + HIFU treatment most efficiently activates antitumor immune response.

## 4 Discussion

Ultrasound microbubbles have widespread applications in various tumor diagnoses and treatment, including their role in enhancing HIFU treatment [30, 31, 32, 33]. USMI by leveraging targeted microbubbles not only offers precise ultrasound imaging for therapy guidance but also effectively amplifies the cavitation effect of HIFU treatment, thereby enhancing therapeutic efficacy [16, 34, 35, 36]. However, challenges in clinical translation often arise due to the unique materials and intricate manufacturing processes [37]. In our study, we utilized an industrially produced platform‐targeted microbubble to enhance the efficacy of HIFU treatment for liver cancer. This type of microbubble, extensively researched for ultrasound molecular imaging diagnosis, has entered clinical or preclinical stages [22, 23, 24, 38], substantially reducing the difficulty of clinical translation.

Our results demonstrate that B7‐H3‐targeted microbubbles enable specific imaging of hepatocellular carcinoma and further exhibit prolonged retention within tumor tissue, providing a longer time window for enhancing HIFU ablation. Compared to the I‐MBs group, the T‐MBs group displayed higher overall contrast intensity and longer duration in the tumor tissue. The high expression of B7‐H3 receptors on the endothelium of blood vessels within liver cancer tissue [28] provides a molecularly targeted enhancement phase in T‐MBs, which is valuable for diagnosis. At 4 min postinjection, T‐MBs and I‐MBs displayed the most significant intensity difference in the tumor region. Therefore, we initiated HIFU treatment at this time point to compare their enhanced effect on HIFU therapy.

Under HIFU irradiation, microbubbles significantly enhance the mechanical effects of HIFU through their inertial cavitation. Specifically, when HIFU ultrasonic waves interact with microbubbles, the bubbles undergo rapid expansion and violent collapse, ultimately leading to rupture. This rupture, caused by inertial cavitation, generates microjets and high shear forces, effectively disrupting the structure of tumor cells [39, 40]. Additionally, the rupture of the microbubbles may also result in a certain degree of heat deposition. GSCs serve as a reliable indicator of coagulation necrosis in HIFU treatment [41]. In our study, we employed gradually decreasing acoustic power to compare the occurrence of GSCs with and without microbubbles. The presence of microbubbles resulted in significant GSCs at an acoustic power of 70 W, while HIFU treatment alone produced GSCs only at an intensity of 90 W. Therefore, T‐MBs effectively reduced the acoustic power required for HIFU during tumor ablation treatment, minimizing the risk of complications caused by damaging normal tissue. Additionally, various assessments, including the immediate evaluation of tumor microbubble perfusion through post‐treatment ultrasound imaging, changes in tumor volume, and survival observation of tumor‐bearing mice, indicated better therapeutic outcomes in T‐MBs + HIFU group compared to other groups. H&E staining for cell apoptosis and proliferation confirmed the most significant enhancement of HIFU‐mediated tumor ablation in the T‐MBs + HIFU group. The I‐MBs + HIFU group also demonstrated an enhanced antitumor effect compared to the HIFU treatment alone. Given the much longer HIFU treatment duration required for tumor ablation in clinical use, it can be speculated that targeted microbubble would provide a greater advantage in terms of microbubble quantity and retention time in the tumor region.

In our study, we observed an acute inflammatory reaction after HIFU treatment. The concentration of cytokines, including IFN‐γ, TNF‐α, and IL‐6 in the serum, increased significantly in the T‐MBs + HIFU group 1 day after treatment. These cytokines can activate DCs and CD8+ T cells to induce antitumor immunity [42, 43, 44]. Meanwhile, IL‐10, known to promote exhaustion of CD8+ tumor‐infiltrating lymphocytes and suppress the activation of natural killer cells [45, 46], was the lowest in the T‐MBs + HIFU group. The study by Avinash Eranki et al. [10] also confirmed that HIFU can induce an increase in pro‐inflammatory factors in the serum. The difference is that their study found that the concentration of pro‐inflammatory factors in the serum was highest on the third day after treatment. The possible reason is that the HIFU parameters used in our study were thermal ablation parameters, and the tumor was fully covered, while they used a lower duty cycle mechanical HIFU and only 2% of the tumor area was treated. We speculate that the larger range of treatment coverage and extensive coagulation necrosis may cause a faster systemic acute inflammatory reaction. The level of pro‐inflammatory factors in T‐MBs + HIFU group in tumor tissue was higher than that in other groups on the third or seventh day after treatment, showing a gradual infiltration of immune response. The possible reason is that HIFU causes the occlusion of tumor neovascularization due to coagulation necrosis or thrombosis [47, 48], resulting in slow infiltration of inflammatory cells and release of pro‐inflammatory factors. Additionally, the decrease in the expression of Hsp70 and the increase of CRT in the combined treatment group revealed the MBs‐enhanced mechanical effect. HIFU can produce a large amount of tumor cell debris through thermal and mechanical destruction, thereby promoting tumor antigen exposure and inducing an antitumor immune response [49, 50]. Studies in mouse models indicate that the mechanical effect of HIFU may induce a stronger antitumor immune response compared to its thermal effect [51, 52]. In our experiments, by introducing T‐MBs as cavitation nuclei to lower the threshold for cavitation [53], the mechanical effect of HIFU was significantly enhanced and immune activation was then augmented.

Despite these promising results, our study has some limitations. Firstly, the tumor volume in the animal model we used is relatively small, potentially undermining the ability to capture differences in treatment efficacy among the different groups. Secondly, the effect of different parameters of HIFU treatment, such as energy selection and duty cycle, and the proportion of tumor ablation volume on the activation of antitumor immune response, was not considered. It is valuable to explore different ultrasound parameters for T‐MBs combined with HIFU to ablate tumors and enhance antitumor immune response.

## 5 Conclusion

In summary, ultrasound molecular imaging is achieved in liver cancer in mice models using platform‐based microbubbles targeting to vascular endothelial B7‐H3 receptors. T‐MBs effectively enhanced the efficacy of HIFU in ablating tumors. Compared to the HIFU alone and I‐MBs + HIFU treatment, the T‐MBs + HIFU treatment demonstrated better tumor ablation and growth inhibition effects, as well as the progressively intensified antitumor immune response. These findings provide a valuable preclinical research foundation for the clinical translation of targeted microbubbles in combination with HIFU for tumor treatment.

## Author Contributions

**Xialin Xiong:** data curation (equal), formal analysis (equal), funding acquisition (equal), methodology (equal), writing – original draft (equal). **Hang Zhou:** data curation (equal), formal analysis (equal), funding acquisition (equal), methodology (equal), writing – original draft (equal). **Xinzhi Xu:** data curation (equal), formal analysis (equal), methodology (equal). **Qihuan Fu:** formal analysis (equal), methodology (equal). **Yujie Wan:** data curation (equal), formal analysis (equal), methodology (equal). **Yuting Cao:** data curation (equal), methodology (equal). **Rui Tang:** data curation (equal), methodology (equal). **Fang Li:** conceptualization (equal), funding acquisition (equal), methodology (equal), supervision (equal). **Jun Zhang:** conceptualization (equal), data curation (equal), formal analysis (equal), methodology (equal), resources (equal). **Pan Li:** conceptualization (lead), funding acquisition (lead), project administration (lead), supervision (lead), writing – review and editing (lead).

## Ethics Statement

Animal experimental procedures strictly adhered to the guidelines set by the Animal Ethics Committee of the Second Affiliated Hospital of Chongqing Medical University (Ethical approval number: (2023) 594).

## Conflicts of Interest

The authors declare no conflicts of interest.

## Supporting information


**Figure S1.** Characterization of microbubbles. (A) Laser scanning confocal microscope (LSCM) of microbubbles and FITC‐labeled secondary antibody connected microbubbles. (B) Flow cytometry analysis of the connection efficiency of FITC to microbubbles.

**Figure S2.** In vivo ultrasound molecular imaging of livers. (A) Ultrasound molecular imaging of livers with T‐MBs and I‐MBs in vivo. (B) Grayscale quantitative analysis of A, respectively (*n* = 3).

**Figure S3.** The post‐treatment changes in body weight of the mice. The body weights of the mice in all groups remained consistent.

**Figure S4.** Cytokine (IFN‐γ, TNF‐α, IL‐6, and IL‐10) concentrations in serum samples from various groups of mice by ELISA assay (*n* = 3, *p < 0.05, **p < 0.01, ***p < 0.001, ****p < 0.0001).

**Figure S5.** Cytokine (IFN‐γ, TNF‐α, IL‐6, and IL‐10) concentrations in tumor tissue samples from various groups of mice by ELISA assay (*n* = 3, *p < 0.05, **p < 0.01, ***p < 0.001).

**Figure S6.** (A) Immunofluorescence analysis of M1 cells (CD86+, red fluorescence), and (B) M2 cells (CD206+, red fluorescence) in tumor tissues (Scale bar: 50 μm).

## Data Availability

All the raw data are available by contacting the main corresponding author.
