# Supplementary figures and images for: Ultrasound Molecular Imaging Enhances High‐Intensity Focused Ultrasound Ablation on Liver Cancer With B7‐H3‐Targeted Microbubbles
Source: Cancer Med. 2024 Oct 21;13(20):e70341. doi: 10.1002/cam4.70341 (PMC11492419; doi:10.1002/cam4.70341)

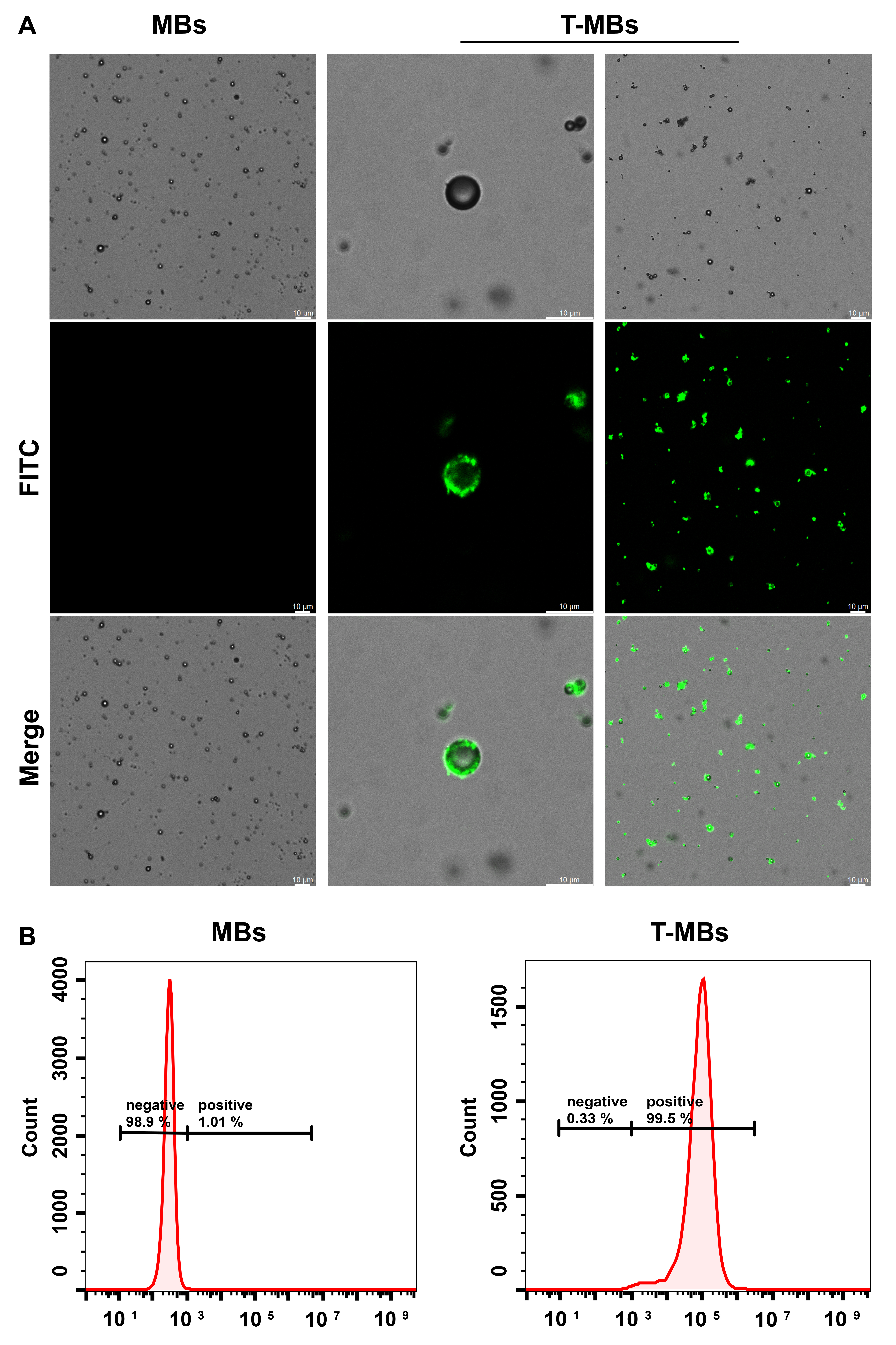

Supplement: Supplementary file 1 — Figure S1. Characterization of microbubbles. (A) Laser scanning confocal microscope (LSCM) of microbubbles and FITC‐labeled secondary antibody connected microbubbles. (B) Flow cytometry analysis of the connection efficiency of FITC to microbubbles. [file CAM4-13-e70341-s003.jpg]

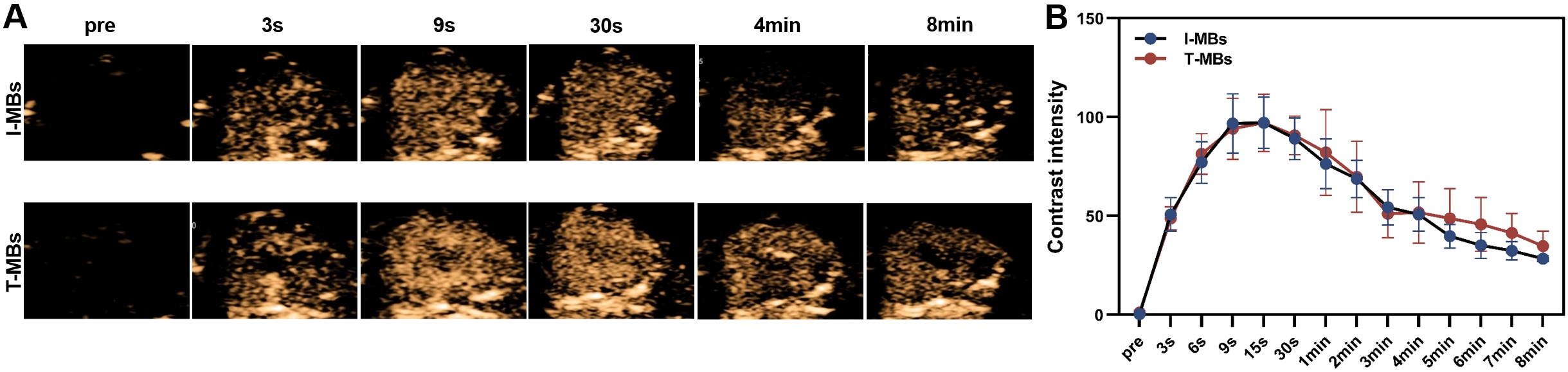

Supplement: Supplementary file 2 — Figure S2. In vivo ultrasound molecular imaging of livers. (A) Ultrasound molecular imaging of livers with T‐MBs and I‐MBs in vivo. (B) Grayscale quantitative analysis of A, respectively (n = 3). [file CAM4-13-e70341-s004.jpg]

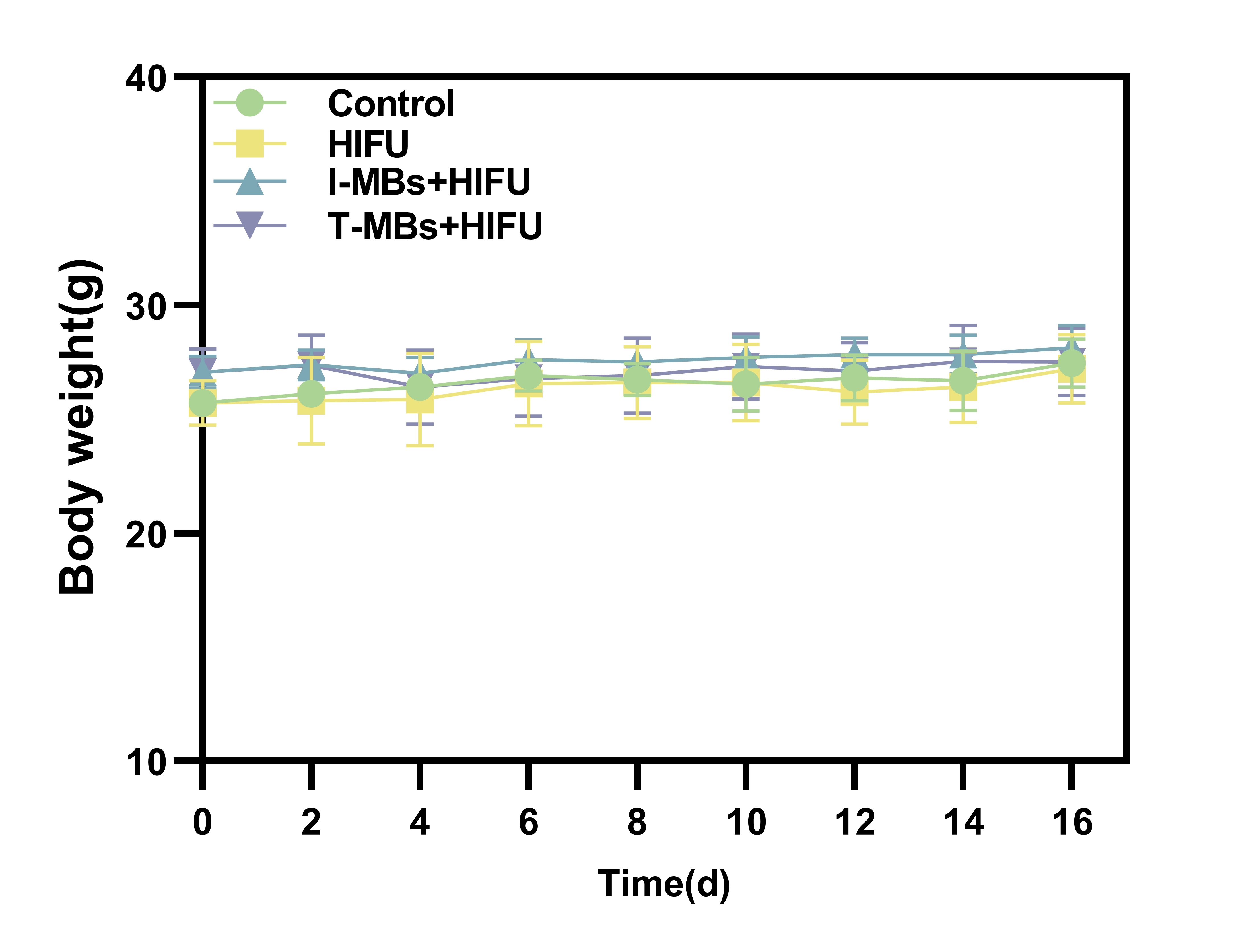

Supplement: Supplementary file 3 — Figure S3. The post‐treatment changes in body weight of the mice. The body weights of the mice in all groups remained consistent. [file CAM4-13-e70341-s006.tif]

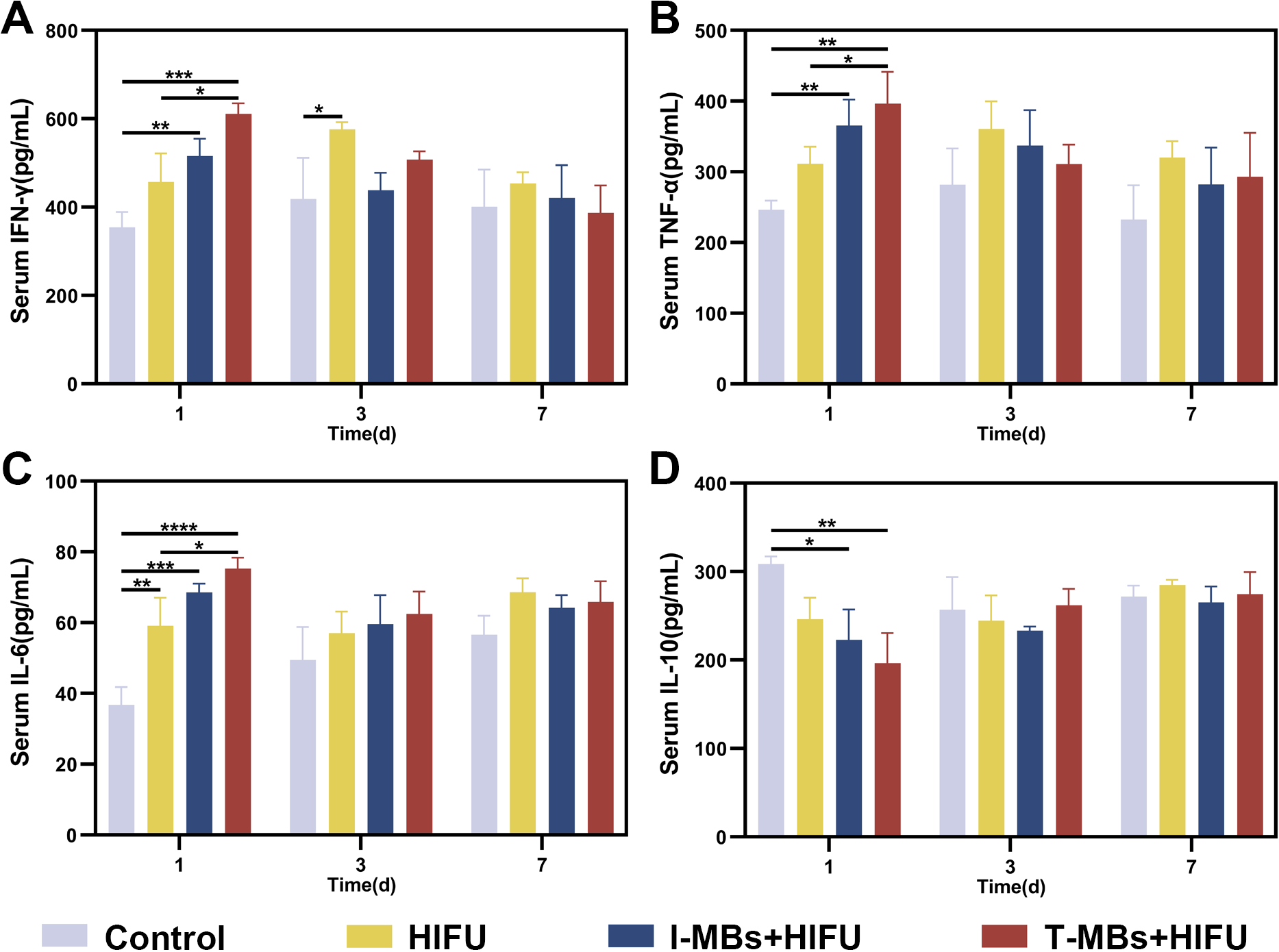

Supplement: Supplementary file 4 — Figure S4. Cytokine (IFN‐γ, TNF‐α, IL‐6, and IL‐10) concentrations in serum samples from various groups of mice by ELISA assay (n = 3, *p < 0.05, **p < 0.01, ***p < 0.001, ****p < 0.0001). [file CAM4-13-e70341-s001.tif]

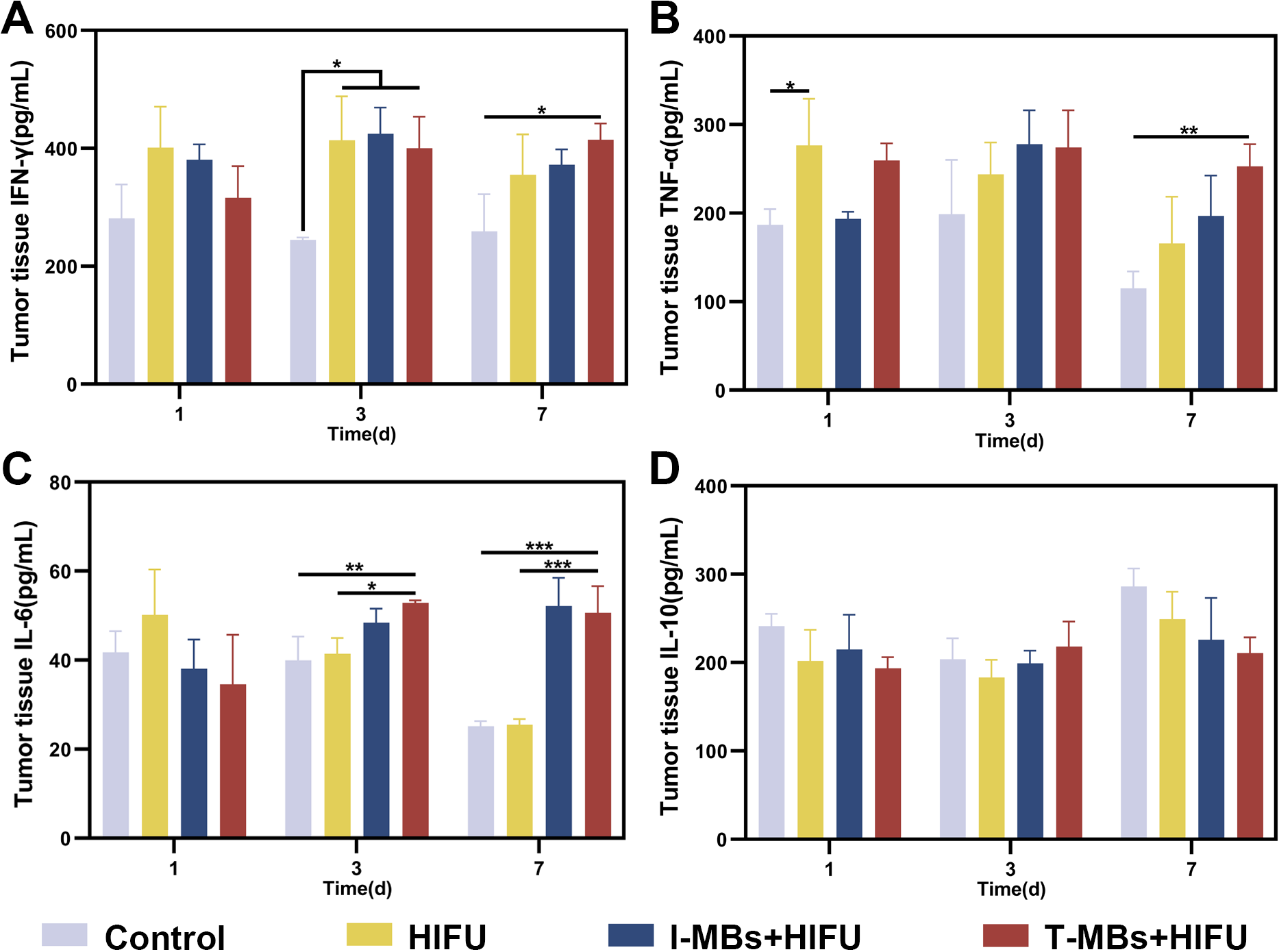

Supplement: Supplementary file 5 — Figure S5. Cytokine (IFN‐γ, TNF‐α, IL‐6, and IL‐10) concentrations in tumor tissue samples from various groups of mice by ELISA assay (n = 3, *p < 0.05, **p < 0.01, ***p < 0.001). [file CAM4-13-e70341-s002.tif]

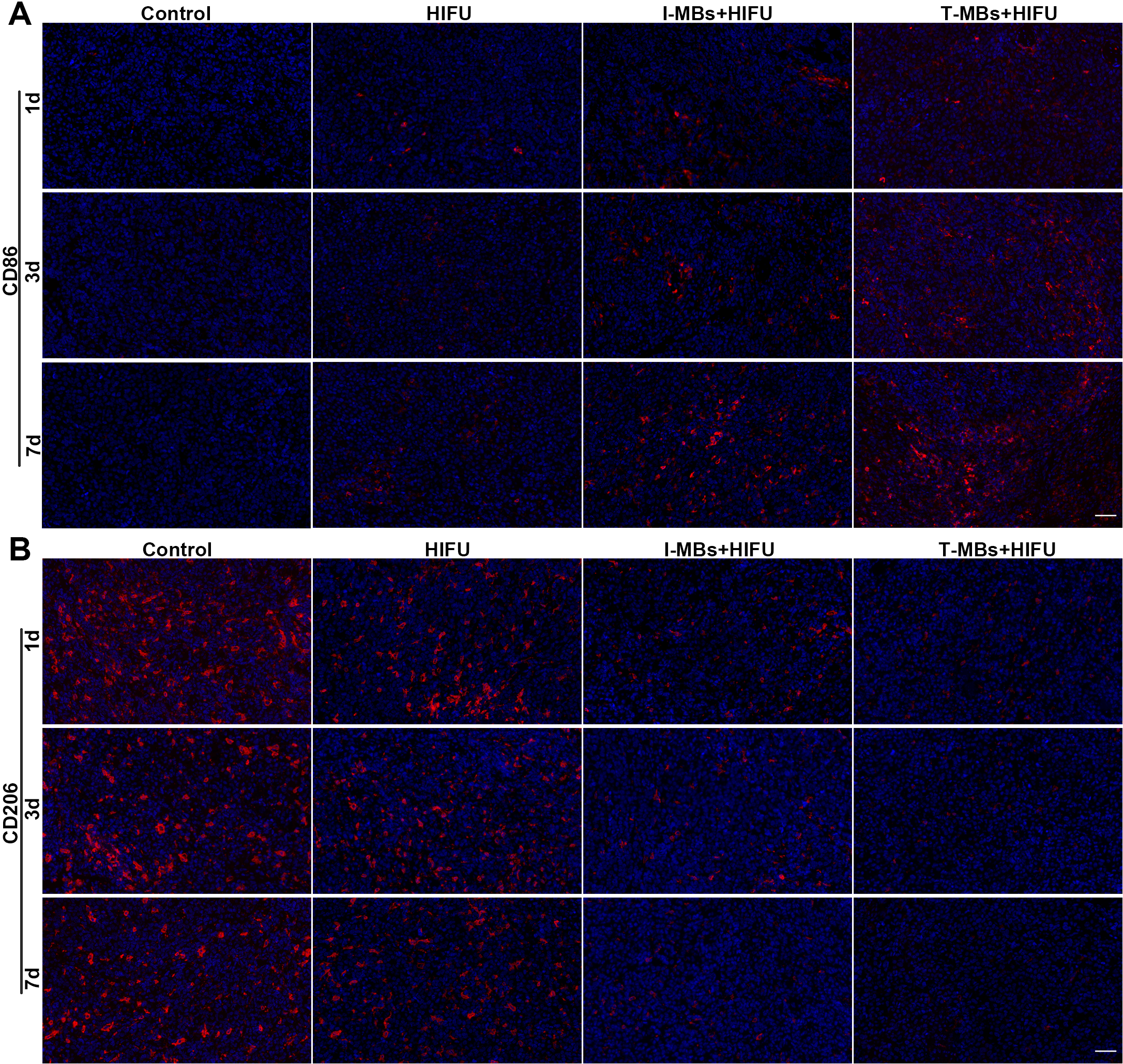

Supplement: Supplementary file 6 — Figure S6. (A) Immunofluorescence analysis of M1 cells (CD86+, red fluorescence), and (B) M2 cells (CD206+, red fluorescence) in tumor tissues (Scale bar: 50 μm). [file CAM4-13-e70341-s005.tif]
